# *N*-(2-Hydroxy­ethyl)-*N*-(tricyclo­[3.3.1.1^3,7^]dec-2-yl)benzamide

**DOI:** 10.1107/S1600536808013469

**Published:** 2008-05-10

**Authors:** Grant A. Boyle, Thavendran Govender, Hendrik G. Kruger, Oluseye K. Onajole

**Affiliations:** a School of Chemistry, University of KwaZulu-Natal, Durban 4000, South Africa; b School of Pharmacy and Pharmacology, University of KwaZulu-Natal, Durban 4000, South Africa

## Abstract

The title adamantane derivative, C_19_H_25_NO_2_, was synthesized as part of a study into potential anti­tuberculosis agents. The adamantane skeleton displays shorter than normal C—C bond lengths ranging between 1.5230 (15) and 1.5329 (16) Å. The structure displays O—H⋯O hydrogen bonding and an inter­digitated layered packing structure with distinct hydro­philic and hydro­phobic regions.

## Related literature

For related literature, see: Bogatcheva *et al.* (2006 ▶); Jacobson *et al.* (1987 ▶); Lee *et al.*, (2003 ▶)
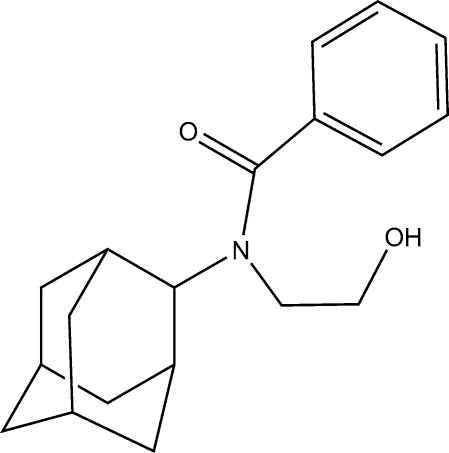


## Experimental

### 

#### Crystal data

C_19_H_25_NO_2_
*M* *_r_* = 299.40
Monoclinic, 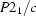

*a* = 11.4248 (3) Å
*b* = 16.0902 (4) Å
*c* = 8.7211 (2) Å
β = 107.9030 (10)°
*V* = 1525.55 (7) Å^3^
*Z* = 4
Mo *K*α radiation
μ = 0.08 mm^−1^
*T* = 173 (2) K
0.44 × 0.33 × 0.16 mm

#### Data collection

Bruker APEXII CCD area-detector diffractometer
Absorption correction: none
19272 measured reflections
3684 independent reflections
2861 reflections with *I* > 2σ(*I*)
*R*_int_ = 0.050

#### Refinement

*R*[*F* ^2^ > 2σ(*F* ^2^)] = 0.041
*wR*(*F* ^2^) = 0.109
*S* = 1.12
3684 reflections
200 parameters
H-atom parameters constrained
Δρ_max_ = 0.29 e Å^−3^
Δρ_min_ = −0.22 e Å^−3^

### 

Data collection: *APEX2* (Bruker, 2005 ▶); cell refinement: *SAINT-Plus* (Bruker, 1999 ▶); data reduction: *SAINT-Plus*; program(s) used to solve structure: *SHELXTL* (Sheldrick, 2008 ▶); program(s) used to refine structure: *SHELXTL*; molecular graphics: *Mercury* (Macrae *et al.*, 2006 ▶) and *WinGX* (Farrugia, 1999 ▶); software used to prepare material for publication: *SHELXTL* and *PLATON* (Spek, 2003 ▶).

## Supplementary Material

Crystal structure: contains datablocks I, global. DOI: 10.1107/S1600536808013469/fl2199sup1.cif

Structure factors: contains datablocks I. DOI: 10.1107/S1600536808013469/fl2199Isup2.hkl

Additional supplementary materials:  crystallographic information; 3D view; checkCIF report

## Figures and Tables

**Table 1:** Hydrogen-bond geometry (Å, °)

*D*—H⋯*A*	*D*—H	H⋯*A*	*D*⋯*A*	*D*—H⋯*A*
O1—H1*A*⋯O2^i^	0.84	1.96	2.7735 (12)	164

Symmetry code: (i) 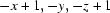.

**Fig. 1.**
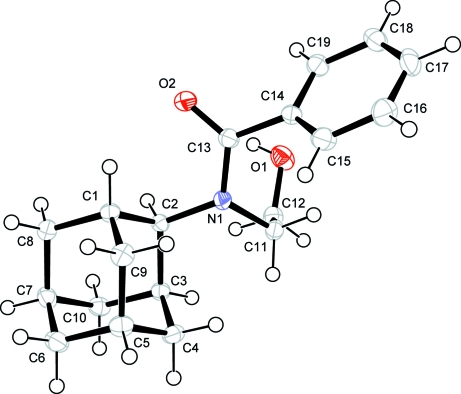
ORTEP diagram showing displacement ellipsoids at the 50% probability level.

**Fig. 2.**
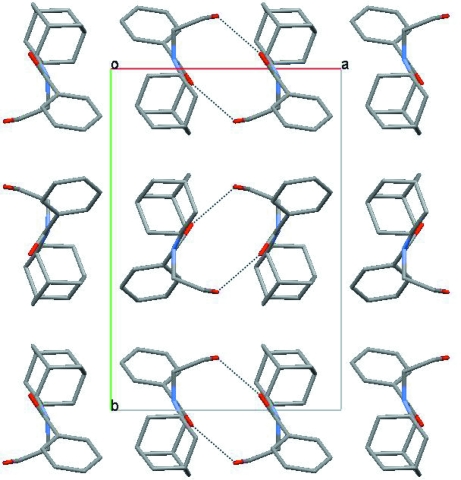
Diagram of the packing viewed down the c axis showing the layered structure and intermolecular hydrogen bonding. Hydrogen atoms have been omitted for clarity.

## supplementary crystallographic information

### Comment

Adamantane derivatives have been the subject of much investigation as potential anti-tubercolosis compounds (Bogatcheva *et al.*, 2006, Lee *et al.*, 2003). The novel compound (I) was also synthesized in order to investigate the biological activities of cage amino-alcohol compounds as potential anti-tuberculosis agents. The molecule (I) consists of a polycyclic (lipophilic) hydrocarbon skeleton with polar amine and hydroxyl units (Fig. 1).

The molecule exhibits some C—C bonds that are significantly shorter than the expected C—C bond length of 1.54 Å. These bonds range between 1.5230 (15) Å for C7—C8 to 1.5329 (16) Å for C1—C8 in the adamantane skeleton.

The structure exhibits intermolecular hydrogen bonding interactions between O1 and O2 of adjacent molecules (Fig. 2). There is also a complex network of short contacts between the molecules which result in an interdigitated, layered structure showing distinct hydrophilic and hydrophobic regions (Fig. 2). The hydrophobic region consists of the adamantane skeleton while the hydrophilic layer consists of the polar amine and hydroxyl units.

### Experimental

To a stirred solution of 2-(tricyclo[3.3.1.13,7]dec-2-ylamino)-ethanol (1 g, 5.1 mmol) in dichloromethane (30 ml) was added dropwise over 45 minutes a solution of benzoyl chloride (590ul, 5.1 mmol) dissolved in 50 ml of dichloromethane under a nitrogen atmosphere at zero degrees using an external ice salt bath. The reaction was allowed to stir overnight (Jacobson *et al.*, 1987) and then filtered to remove the HCl salt  and the solvent was removed *in vacuo*. The mixture was re-crystallized from methanol to obtain the title compound (I) (0.91 g, 60%) as a colourless microcrystalline solid.

### Refinement

Hydrogen atoms were first located in the difference map then positioned geometrically, and allowed to ride on their respective parent atoms, with bond lengths of 0.99 Å (CH_2_), 1.00 Å (Methine CH), 0.95 Å (Ar—CH) or 0.84 Å (OH). Isotropic displacement parameters for these atoms were set equal to 1.2 (CH_2_ and CH), or 1.5 (OH) times *U_eq_* of the parent atom.

### Crystal data

**Table:**

C_19_H_25_NO_2_	*F*_000_ = 648
*M**_r_* = 299.40	*D*_x_ = 1.304 Mg m^−^^3^
Monoclinic, *P*2_1_/*c*	Mo *K*α radiation λ = 0.71073 Å
Hall symbol: -P 2ybc	Cell parameters from 5317 reflections
*a* = 11.4248 (3) Å	θ = 2.3–28.2º
*b* = 16.0902 (4) Å	µ = 0.08 mm^−^^1^
*c* = 8.7211 (2) Å	*T* = 173 (2) K
β = 107.9030 (10)º	Block, colourless
*V* = 1525.55 (7) Å^3^	0.44 × 0.33 × 0.16 mm
*Z* = 4	

### Data collection

**Table:**

Bruker SMART CCD area-detector diffractometer	2861 reflections with *I* > 2σ(*I*)
Radiation source: fine-focus sealed tube	*R*_int_ = 0.050
Monochromator: graphite	θ_max_ = 28.0º
*T* = 173(2) K	θ_min_ = 1.9º
φ and ω scans	*h* = −15→15
Absorption correction: none	*k* = −21→21
19272 measured reflections	*l* = −11→11
3684 independent reflections	

### Refinement

**Table:**

Refinement on *F*^2^	Secondary atom site location: difference Fourier map
Least-squares matrix: full	Hydrogen site location: inferred from neighbouring sites
*R*[*F*^2^ > 2σ(*F*^2^)] = 0.041	H-atom parameters constrained
*wR*(*F*^2^) = 0.109	*w* = 1/[σ^2^(*F*_o_^2^) + (0.0562*P*)^2^] where *P* = (*F*_o_^2^ + 2*F*_c_^2^)/3
*S* = 1.12	(Δ/σ)_max_ = 0.001
3684 reflections	Δρ_max_ = 0.29 e Å^−^^3^
200 parameters	Δρ_min_ = −0.22 e Å^−^^3^
Primary atom site location: structure-invariant direct methods	Extinction correction: none

### Special details

**Table:**

Geometry. All e.s.d.'s (except the e.s.d. in the dihedral angle between two l.s. planes) are estimated using the full covariance matrix. The cell e.s.d.'s are taken into account individually in the estimation of e.s.d.'s in distances, angles and torsion angles; correlations between e.s.d.'s in cell parameters are only used when they are defined by crystal symmetry. An approximate (isotropic) treatment of cell e.s.d.'s is used for estimating e.s.d.'s involving l.s. planes.
Refinement. Refinement of *F*^2^ against ALL reflections. The weighted *R*-factor *wR* and goodness of fit *S* are based on *F*^2^, conventional *R*-factors *R* are based on *F*, with *F* set to zero for negative *F*^2^. The threshold expression of *F*^2^ > σ(*F*^2^) is used only for calculating *R*-factors(gt) *etc*. and is not relevant to the choice of reflections for refinement. *R*-factors based on *F*^2^ are statistically about twice as large as those based on *F*, and *R*- factors based on ALL data will be even larger.

### Fractional atomic coordinates and isotropic or equivalent isotropic displacement parameters (Å^2^)

**Table:**

	*x*	*y*	*z*	*U*_iso_*/*U*_eq_	
C1	0.26872 (10)	0.12522 (7)	0.22178 (13)	0.0191 (2)	
H1	0.2797	0.1396	0.3369	0.023*	
C2	0.32356 (10)	0.03827 (6)	0.21268 (13)	0.0170 (2)	
H2	0.4145	0.0414	0.2656	0.020*	
C3	0.30029 (10)	0.01726 (7)	0.03325 (13)	0.0179 (2)	
H3	0.3322	−0.0398	0.0239	0.021*	
C4	0.16309 (10)	0.02106 (7)	−0.06201 (14)	0.0215 (3)	
H4A	0.1512	0.0068	−0.1762	0.026*	
H4B	0.1172	−0.0198	−0.0177	0.026*	
C5	0.11394 (11)	0.10846 (7)	−0.05095 (14)	0.0226 (3)	
H5	0.0244	0.1109	−0.1128	0.027*	
C6	0.18444 (11)	0.17132 (7)	−0.12046 (14)	0.0250 (3)	
H6A	0.1522	0.2279	−0.1143	0.030*	
H6B	0.1728	0.1583	−0.2352	0.030*	
C7	0.32164 (11)	0.16808 (7)	−0.02533 (14)	0.0220 (3)	
H7	0.3678	0.2092	−0.0707	0.026*	
C8	0.33836 (11)	0.18828 (7)	0.15074 (14)	0.0212 (3)	
H8A	0.3071	0.2449	0.1592	0.025*	
H8B	0.4269	0.1869	0.2126	0.025*	
C9	0.13223 (11)	0.12772 (7)	0.12635 (14)	0.0225 (3)	
H9A	0.0986	0.1835	0.1362	0.027*	
H9B	0.0872	0.0864	0.1709	0.027*	
C10	0.36964 (10)	0.08045 (7)	−0.03724 (13)	0.0210 (3)	
H10A	0.4586	0.0778	0.0223	0.025*	
H10B	0.3586	0.0668	−0.1515	0.025*	
C11	0.27216 (11)	−0.11302 (7)	0.24168 (14)	0.0206 (3)	
H11A	0.2282	−0.1153	0.1246	0.025*	
H11B	0.2266	−0.1482	0.2972	0.025*	
C12	0.40094 (11)	−0.14766 (7)	0.27306 (14)	0.0242 (3)	
H12A	0.3964	−0.2032	0.2226	0.029*	
H12B	0.4490	−0.1107	0.2243	0.029*	
C13	0.29089 (10)	−0.01541 (7)	0.45859 (13)	0.0206 (3)	
C14	0.22837 (11)	−0.07451 (7)	0.54113 (13)	0.0206 (2)	
C15	0.10237 (11)	−0.08725 (8)	0.48552 (15)	0.0264 (3)	
H15	0.0542	−0.0607	0.3896	0.032*	
C16	0.04662 (12)	−0.13882 (9)	0.56983 (16)	0.0316 (3)	
H16	−0.0399	−0.1468	0.5324	0.038*	
C17	0.11626 (12)	−0.17845 (8)	0.70758 (15)	0.0299 (3)	
H17	0.0780	−0.2149	0.7635	0.036*	
C18	0.24153 (12)	−0.16534 (7)	0.76451 (15)	0.0270 (3)	
H18	0.2895	−0.1923	0.8601	0.032*	
C19	0.29721 (11)	−0.11306 (7)	0.68239 (14)	0.0231 (3)	
H19	0.3833	−0.1034	0.7229	0.028*	
N1	0.27326 (8)	−0.02630 (5)	0.29804 (11)	0.0183 (2)	
O1	0.46035 (8)	−0.15413 (6)	0.44118 (10)	0.0311 (2)	
H1A	0.5219	−0.1226	0.4676	0.047*	
O2	0.34988 (8)	0.04282 (5)	0.53714 (10)	0.0301 (2)	

### Atomic displacement parameters (Å^2^)

**Table:**

	*U*^11^	*U*^22^	*U*^33^	*U*^12^	*U*^13^	*U*^23^
C1	0.0225 (6)	0.0194 (5)	0.0167 (5)	−0.0003 (4)	0.0081 (5)	−0.0023 (4)
C2	0.0180 (5)	0.0183 (5)	0.0157 (5)	−0.0021 (4)	0.0064 (4)	0.0004 (4)
C3	0.0201 (6)	0.0183 (5)	0.0166 (5)	−0.0001 (4)	0.0075 (4)	−0.0018 (4)
C4	0.0212 (6)	0.0250 (6)	0.0177 (6)	−0.0035 (5)	0.0052 (5)	−0.0039 (4)
C5	0.0161 (6)	0.0281 (6)	0.0217 (6)	0.0016 (5)	0.0030 (5)	−0.0006 (5)
C6	0.0282 (7)	0.0261 (6)	0.0199 (6)	0.0028 (5)	0.0060 (5)	0.0034 (5)
C7	0.0244 (6)	0.0218 (6)	0.0214 (6)	−0.0014 (5)	0.0094 (5)	0.0032 (5)
C8	0.0235 (6)	0.0186 (5)	0.0218 (6)	−0.0019 (5)	0.0071 (5)	−0.0007 (4)
C9	0.0208 (6)	0.0240 (6)	0.0248 (6)	0.0013 (5)	0.0102 (5)	−0.0015 (5)
C10	0.0193 (6)	0.0269 (6)	0.0183 (6)	−0.0003 (5)	0.0082 (5)	0.0000 (5)
C11	0.0237 (6)	0.0178 (5)	0.0220 (6)	−0.0031 (4)	0.0094 (5)	−0.0016 (4)
C12	0.0251 (6)	0.0211 (6)	0.0282 (7)	0.0004 (5)	0.0109 (5)	0.0004 (5)
C13	0.0208 (6)	0.0225 (6)	0.0182 (6)	−0.0011 (5)	0.0054 (5)	0.0010 (4)
C14	0.0248 (6)	0.0206 (6)	0.0183 (5)	−0.0017 (5)	0.0097 (5)	−0.0022 (4)
C15	0.0242 (6)	0.0327 (7)	0.0222 (6)	−0.0018 (5)	0.0070 (5)	0.0008 (5)
C16	0.0248 (7)	0.0387 (7)	0.0349 (7)	−0.0082 (5)	0.0142 (6)	−0.0039 (6)
C17	0.0386 (8)	0.0254 (6)	0.0340 (7)	−0.0043 (6)	0.0233 (6)	0.0009 (5)
C18	0.0368 (7)	0.0234 (6)	0.0246 (6)	0.0045 (5)	0.0150 (6)	0.0028 (5)
C19	0.0226 (6)	0.0248 (6)	0.0233 (6)	0.0002 (5)	0.0091 (5)	0.0002 (5)
N1	0.0212 (5)	0.0171 (5)	0.0178 (5)	−0.0024 (4)	0.0078 (4)	−0.0006 (4)
O1	0.0250 (5)	0.0372 (5)	0.0302 (5)	0.0001 (4)	0.0070 (4)	0.0092 (4)
O2	0.0386 (5)	0.0317 (5)	0.0189 (4)	−0.0140 (4)	0.0072 (4)	−0.0028 (4)

### Geometric parameters (Å, °)

**Table:**

C1—C9	1.5258 (15)	C9—H9B	0.9900
C1—C8	1.5329 (16)	C10—H10A	0.9900
C1—C2	1.5450 (15)	C10—H10B	0.9900
C1—H1	1.0000	C11—N1	1.4783 (14)
C2—N1	1.4922 (14)	C11—C12	1.5174 (16)
C2—C3	1.5420 (15)	C11—H11A	0.9900
C2—H2	1.0000	C11—H11B	0.9900
C3—C10	1.5293 (15)	C12—O1	1.4175 (14)
C3—C4	1.5330 (15)	C12—H12A	0.9900
C3—H3	1.0000	C12—H12B	0.9900
C4—C5	1.5283 (16)	C13—O2	1.2319 (13)
C4—H4A	0.9900	C13—N1	1.3632 (14)
C4—H4B	0.9900	C13—C14	1.4996 (16)
C5—C9	1.5270 (16)	C14—C15	1.3858 (16)
C5—C6	1.5295 (17)	C14—C19	1.3876 (16)
C5—H5	1.0000	C15—C16	1.3867 (18)
C6—C7	1.5325 (16)	C15—H15	0.9500
C6—H6A	0.9900	C16—C17	1.3766 (19)
C6—H6B	0.9900	C16—H16	0.9500
C7—C8	1.5230 (15)	C17—C18	1.3793 (18)
C7—C10	1.5280 (16)	C17—H17	0.9500
C7—H7	1.0000	C18—C19	1.3804 (16)
C8—H8A	0.9900	C18—H18	0.9500
C8—H8B	0.9900	C19—H19	0.9500
C9—H9A	0.9900	O1—H1A	0.8400
			
C9—C1—C8	109.43 (9)	C1—C9—H9A	109.6
C9—C1—C2	111.00 (9)	C5—C9—H9A	109.6
C8—C1—C2	108.03 (9)	C1—C9—H9B	109.6
C9—C1—H1	109.5	C5—C9—H9B	109.6
C8—C1—H1	109.5	H9A—C9—H9B	108.1
C2—C1—H1	109.5	C7—C10—C3	110.19 (9)
N1—C2—C3	112.39 (9)	C7—C10—H10A	109.6
N1—C2—C1	112.35 (9)	C3—C10—H10A	109.6
C3—C2—C1	107.73 (9)	C7—C10—H10B	109.6
N1—C2—H2	108.1	C3—C10—H10B	109.6
C3—C2—H2	108.1	H10A—C10—H10B	108.1
C1—C2—H2	108.1	N1—C11—C12	112.21 (9)
C10—C3—C4	108.99 (9)	N1—C11—H11A	109.2
C10—C3—C2	108.25 (9)	C12—C11—H11A	109.2
C4—C3—C2	111.70 (9)	N1—C11—H11B	109.2
C10—C3—H3	109.3	C12—C11—H11B	109.2
C4—C3—H3	109.3	H11A—C11—H11B	107.9
C2—C3—H3	109.3	O1—C12—C11	110.06 (10)
C5—C4—C3	109.69 (9)	O1—C12—H12A	109.6
C5—C4—H4A	109.7	C11—C12—H12A	109.6
C3—C4—H4A	109.7	O1—C12—H12B	109.6
C5—C4—H4B	109.7	C11—C12—H12B	109.6
C3—C4—H4B	109.7	H12A—C12—H12B	108.2
H4A—C4—H4B	108.2	O2—C13—N1	123.49 (10)
C9—C5—C4	108.18 (9)	O2—C13—C14	118.37 (10)
C9—C5—C6	110.02 (10)	N1—C13—C14	118.02 (10)
C4—C5—C6	109.57 (10)	C15—C14—C19	119.26 (11)
C9—C5—H5	109.7	C15—C14—C13	121.52 (10)
C4—C5—H5	109.7	C19—C14—C13	119.08 (10)
C6—C5—H5	109.7	C14—C15—C16	120.01 (12)
C5—C6—C7	109.79 (9)	C14—C15—H15	120.0
C5—C6—H6A	109.7	C16—C15—H15	120.0
C7—C6—H6A	109.7	C17—C16—C15	120.22 (12)
C5—C6—H6B	109.7	C17—C16—H16	119.9
C7—C6—H6B	109.7	C15—C16—H16	119.9
H6A—C6—H6B	108.2	C16—C17—C18	120.05 (12)
C8—C7—C10	109.21 (9)	C16—C17—H17	120.0
C8—C7—C6	109.21 (10)	C18—C17—H17	120.0
C10—C7—C6	108.86 (9)	C17—C18—C19	119.94 (12)
C8—C7—H7	109.8	C17—C18—H18	120.0
C10—C7—H7	109.8	C19—C18—H18	120.0
C6—C7—H7	109.8	C18—C19—C14	120.48 (11)
C7—C8—C1	110.27 (9)	C18—C19—H19	119.8
C7—C8—H8A	109.6	C14—C19—H19	119.8
C1—C8—H8A	109.6	C13—N1—C11	116.51 (9)
C7—C8—H8B	109.6	C13—N1—C2	117.71 (9)
C1—C8—H8B	109.6	C11—N1—C2	117.16 (9)
H8A—C8—H8B	108.1	C12—O1—H1A	109.5
C1—C9—C5	110.23 (9)		
			
C9—C1—C2—N1	−67.42 (12)	C4—C3—C10—C7	60.22 (11)
C8—C1—C2—N1	172.61 (9)	C2—C3—C10—C7	−61.46 (11)
C9—C1—C2—C3	56.91 (12)	N1—C11—C12—O1	65.78 (12)
C8—C1—C2—C3	−63.06 (11)	O2—C13—C14—C15	121.39 (13)
N1—C2—C3—C10	−172.53 (9)	N1—C13—C14—C15	−54.82 (15)
C1—C2—C3—C10	63.16 (11)	O2—C13—C14—C19	−54.31 (15)
N1—C2—C3—C4	67.48 (11)	N1—C13—C14—C19	129.49 (11)
C1—C2—C3—C4	−56.83 (11)	C19—C14—C15—C16	−0.74 (18)
C10—C3—C4—C5	−59.62 (12)	C13—C14—C15—C16	−176.43 (11)
C2—C3—C4—C5	59.94 (12)	C14—C15—C16—C17	−0.94 (19)
C3—C4—C5—C9	−60.20 (12)	C15—C16—C17—C18	1.6 (2)
C3—C4—C5—C6	59.75 (12)	C16—C17—C18—C19	−0.61 (19)
C9—C5—C6—C7	58.95 (12)	C17—C18—C19—C14	−1.08 (18)
C4—C5—C6—C7	−59.87 (12)	C15—C14—C19—C18	1.75 (17)
C5—C6—C7—C8	−59.44 (12)	C13—C14—C19—C18	177.55 (11)
C5—C6—C7—C10	59.72 (12)	O2—C13—N1—C11	144.02 (12)
C10—C7—C8—C1	−59.05 (12)	C14—C13—N1—C11	−39.99 (14)
C6—C7—C8—C1	59.90 (12)	O2—C13—N1—C2	−2.93 (16)
C9—C1—C8—C7	−59.43 (12)	C14—C13—N1—C2	173.06 (9)
C2—C1—C8—C7	61.54 (11)	C12—C11—N1—C13	−80.57 (12)
C8—C1—C9—C5	58.41 (12)	C12—C11—N1—C2	66.57 (12)
C2—C1—C9—C5	−60.73 (12)	C3—C2—N1—C13	178.49 (9)
C4—C5—C9—C1	61.06 (12)	C1—C2—N1—C13	−59.80 (13)
C6—C5—C9—C1	−58.61 (12)	C3—C2—N1—C11	31.75 (13)
C8—C7—C10—C3	58.96 (12)	C1—C2—N1—C11	153.46 (9)
C6—C7—C10—C3	−60.20 (12)		

### Hydrogen-bond geometry (Å, °)

**Table:**

*D*—H···*A*	*D*—H	H···*A*	*D*···*A*	*D*—H···*A*
O1—H1A···O2^i^	0.84	1.96	2.7735 (12)	164

Symmetry codes: (i) −*x*+1, −*y*, −*z*+1.
